# A set of multiplex panels of microsatellite markers for rapid molecular characterization of rice accessions

**DOI:** 10.1186/1471-2229-7-23

**Published:** 2007-05-21

**Authors:** Marco Pessoa-Filho, André Beló, António AN Alcochete, Paulo HN Rangel, Márcio E Ferreira

**Affiliations:** 1 Departamento de Biologia Celular, IB – Universidade de Brasília (UnB) Campus Universitario, Asa Norte, CEP 70.910-900, Brasilia – DF, Brazil; 2 Embrapa Recursos Genéticos e Biotecnologia, CP 02372, CEP 70.879-970, Brasilia – DF, Brazil; 3 University of Delaware, College of Agriculture and Natural Resources, Department of Plant and Soil Sciences.152 Townsend Hall, 19716 – Newark, USA; 4 Universidade Agostinho Neto, Dep. Biologia, Av. 4 de Fevereiro no 7, Caixa Postal 815, Luanda, Angola; 5 Embrapa Rice and Beans Rodovia Goiania a Nova Veneza, km 12, Fazenda Capivara C.P. 179; 75375-000 Santo Antonio de Goias, GO, Brazil; 6 Universidade Católica de Brasília, CAMPUS II, SGAN Quadra 916, Modulo B, Av. W5 Norte – Brasilia, DF, CEP: 70790-160, Brazil

## Abstract

**Background:**

This study aimed to analyze the efficiency of three new microsatellite multiplex panels, which were designed to evaluate a total of 16 loci of the rice genome, based on single PCR reactions of each panel. A sample of 548 accessions of traditional upland rice landraces collected in Brazil in the last 25 years was genotyped, a database of allelic frequencies was established, estimates of genetic parameters were performed and analysis of genetic structure of the collection was developed.

**Results:**

The three panels yielded a combined matching probability of 6.4 × 10^-21^, polymorphism information content (PIC) of 0.637, and a combined power of exclusion greater than 99.99%. A few samples presented a genetic background of *indica *rice. The 16 SSR loci produced a total of 229 alleles. Gene diversity values averaged 0.667, and PIC values averaged 0.637. Genetic structure analysis of the collection using a Bayesian approach detected three possible major clusters, with an overall *F*_ST _value of 0.177. Important inputs on the knowledge about upland rice germplasm differentiations which happened in Brazil in the last few centuries were also achieved and are discussed.

**Conclusion:**

The three multiplex panels described here represent a powerful tool for rice genetic analysis, offering a rapid and efficient option for rice germplasm characterization. The data gathered demonstrates the feasibility of genotyping extensive germplasm collections using panels of multiplexed microsatellite markers. It contributes to the advancement of research on large scale characterization and management of germplasm banks, as well as identification, protection and assessments of genetic relationship of rice germplasm.

## Background

One of the largest *ex situ *germplasm collections in the world is comprised of rice accessions (*Oryza sativa *L.) [1]. Its two cultivated Asian subspecies, *indica *and *japonica*, have constituted one of the pillars of human diet for thousands of years. In Brazil, rice production and consumption is comparable to that of some Asian countries, and *japonica *rice accounts for 40% of the total rice production, a value above the 20% average observed in other parts of the world [2]. EMBRAPA keeps a germplasm bank of landraces collected all around the country within a 25-year period. Most of these landraces have been collected in villages and isolated rural areas, where cultivated rice has been grown since its introduction in Brazil, centuries ago [3]. They may represent an extraordinary source of genes that control traits of economic importance, such as drought tolerance and resistance to plant pathogens. The great majority of these rice accessions have not been characterized by any means yet. Technical information is limited to field observation, farmer testimony and descriptive data of the collection site. It is, therefore, an ideal material for genetic characterization based on molecular technology. Molecular data would provide a basis for better management and conservation of the collection and could be used as reference for its enhanced use in breeding programs. Of particular interest is the understanding of the genetic structure of the collection and its potential exploitation for cultivar improvement.

Molecular marker technology has proved to be an efficient tool for plant genetic resource characterization, conservation, and management. Among all different classes of molecular markers available for evaluating genetic diversity, microsatellites or simple sequence repeats (SSRs) [4,5] are well known for their potentially high information content and versatility as molecular tools [6]. Thousands of microsatellite markers have been developed for rice research so far, having their chromosomal location and polymorphism levels determined [7,8]. They have been extensively used in various fields such as genetic mapping of economically important traits [8-15], and assessments of the level and structure of genetic diversity in cultivars of interest [16-21].

The use of fluorescently labeled microsatellite marker panels greatly increases the capacity of semiautomated genotyping of a large number of accessions, allowing for a faster and highly informative characterization of genetic resources. Fluorescently-based semiautomated genotyping was first reported for the analysis of restriction fragments [22], and was later adapted for microsatellite analysis [23,24]. In rice genetics, the use of 27 fluorescently labeled markers in four panels for the analysis of rice genetic diversity has been described [25], as well as the development of multiplex panels aiming genetic assessments with a complete coverage of the rice genome [26]. In both studies, however, PCR's were performed individually for each marker and the PCR products mixed before electrophoresis. We believe that one could greatly increase the amount of collected information and decrease labor if PCR of multiple loci is done in a single assay prior to electrophoresis.

In this study, fluorescently labeled microsatellite marker panels for semiautomated genotyping were designed and tested on a large number of rice landraces collected and deposited in the EMBRAPA rice gene bank. Only one PCR per sample was used to amplify alleles at multiple microsatellite loci composing each specific multiplex panel. The obtained data were used to estimate the efficiency of the combined multiplex panels in molecular characterization and cultivar identification. It also allowed for the estimation of genetic diversity parameters, germplasm organization, and for the establishment of a database of allelic frequencies for *japonica *rice landraces collected in Brazil.

## Results

### Genetic background of the rice accessions

Initially, it was necessary to verify if the accessions of rice collected in different parts of Brazil belonged to the same genetic background (*japonica*), as indicated by previous information on each of the accessions. Any genetic parameter estimated with the three multiplex marker panels tested in this study could be affected otherwise. Therefore, pairwise genetic distances among 548 accessions were estimated and Neighbor-Joining analysis suggested that the rice accessions could actually be classified into two main clusters, corresponding to materials with a possible *indica *and *japonica *genetic backgrounds (See additional file 1). However, the great majority of the accessions (~90%) belonged to the major cluster, where no *indica *accessions were included. In order to identify possible accessions which still might have been erroneously identified as *japonica*, a bootstrap analysis of the collected data sets for all possible *indica *samples was performed, so that the relative probabilities of inclusion for these samples in the *japonica *or *indica *gene pools would be obtained [27]. These calculations were performed using the WHICHRUN software v.4.1. The allelic frequencies previously estimated for *japonica *and *indica *cultivars [18] were taken as references and used as baseline input data for the comparisons. The results showed that all 63 samples of the minor cluster (See additional file 1) would be more probably described as possessing an *indica *background, with a minimum probability which was at least 4 orders of magnitude higher than the probability of inclusion of these samples in the *japonica *group. Therefore, a total of 485 accessions of the original collection of 548 rice varieties were classified as *japonica *rice. In the group of 485 *japonica *accessions, 469 upland rice landraces have been collected in the Brazilian territory. It should be clarified that subspecies identification was not the purpose of this study. The definition of the *indica *cluster and its elimination from some of the further analyses (see below) had the main objective of avoiding contamination of accessions possessing a probable *indica *genetic background, what could interfere with the multiplex panel analysis.

### Diversity analysis and multiplex panel efficiency in molecular characterization

The level of polymorphism among the 485 *japonica *accessions detected by the three multiplex panels was estimated by calculating the number of different alleles for each locus, the observed heterozygosity (*H*), gene diversity (*GD*), and PIC values (Table 1). The three panels of 16 SSR markers produced a total of 229 alleles for all loci, ranging from 8 alleles for markers RM420 and RM418 to 26 alleles for marker OG106, with an average number of 14. Gene diversity (*GD*) values averaged 0.667, ranging from a low of 0.041 for RM475 to a high of 0.919 to OG106. PIC values averaged 0.637. The database of allelic frequencies shows that rare alleles (with a frequency < 0.05) comprised 76.8% of all alleles, while intermediate (0.05 < frequency < 0.30) and abundant alleles (frequency > 0.30) comprised 19.3% and 3.9% of all detected alleles, respectively. The matching probability or the probability of identical genotypes was estimated for all combined loci as 6.4 × 10^-21 ^(2.9 × 10^-7 ^from Panel A, 1.03 × 10^-9 ^from Panel B, and 2.1 × 10^-5 ^from Panel C). Finally, the combined power of exclusion of the 16 loci in the three multiplex panels was estimated as being greater than 99.99% (93.35% from Panel A, 99.01% from panel B and 99.69% from Panel C).

### Genetic structure of the germplasm collection

**Figure 1:**
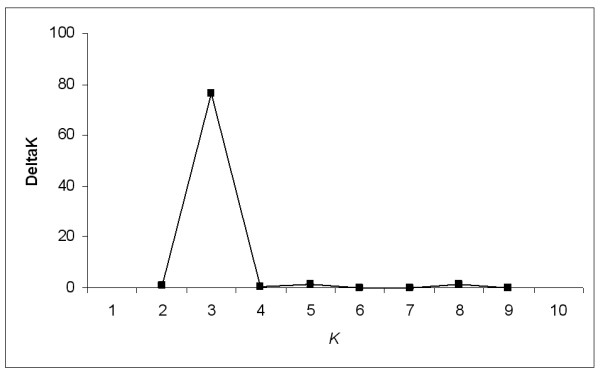
Values of Δ*K*, with its modal value detecting a true *K *of 3 groups (*K *= 3).

The model-based program Structure was used to infer population structure that might be present in this sample of 548 landraces (*indica *and *japonica*) collected in Brazil. Estimated likelihood values for a given *K *in five independent runs were consistent, and increased as the values of *K *increased, a behavior which is expected when factors such as inbreeding and departures from Hardy-Weinberg equilibrium are present [28]. These factors could lead to an overestimation of the number of populations *K*. In order to overcome the difficulty in interpreting which the real value of *K *would be, another *ad hoc *quantity (Δ*K*) was used. It was developed and tested under different simulation routines where real population structure was present [29]. Δ*K *showed to be a good predictor of the uppermost hierarchical level present in a sample, although problems such as its inability in detecting the absence of structure (when *K *= 1) are present. In this study, the highest value of Δ*K *for the 548 accessions was for *K *= 3, with values for other *K'*s being close to zero (Figure 1). Other information provided by Structure, namely the value of α and its behavior, and patterns in the assignment of individuals to different groups led us to choose *K *= 3 for the remaining analyses (data not shown). However, other values of *K *due to the presence of subgroups inside the major groups are possible. Most of the accessions were clearly assigned to a single population following the analysis with *K *= 3 – those which presented more than 70% of their inferred ancestry to a single group – with 73 accessions (approximately 13% of all accessions) identified as admixed. As expected, when those accessions presenting an *indica *genetic background were excluded from the analysis, the 485 *japonica *landraces presented a most probable number of clusters of *K *= 2.

The AMOVA based on the collection of 548 accessions shows that 11.9% of the variation was caused by differences among groups, with the remaining 88.1% being caused by differences within groups. Pairwise *F*_ST _estimates among groups ranged from 0.17 to 0.31, showing that Groups 1 and 3 are those more differentiated from each other (Table 2). Overall *F*_ST _(θ) value was 0.177, indicating a considerable degree of differentiation among the three groups. Overall *F*_IS _and *F*_IT _values were 0.936 and 0.947, respectively, reflecting the effect of inbreeding for a self-pollinating species such as rice. All values are significantly greater than zero (α = 0.05). Pairwise *R*_ST _estimates among groups ranged from 0.193 to 0.371, in agreement with *F*_ST _values regarding greater differences among Groups 1 and 3.

A comparison of the levels of polymorphism in the three defined groups shows how genetic diversity is organized and divided among the inferred populations (Table 2). When only the two groups comprised of *japonica *accessions (groups 1 and 2), with no evidence of an *indica *genetic background are compared, group 2, constituted mostly by accessions from Northeastern Brazil, is the one with higher levels of *GD *and PIC (0.68 and 0.65, respectively). However, group 3, with a much smaller number of accessions, but comprised of those with a probable *indica *genetic background, embodies the highest levels of polymorphism (*GD *= 0.79 and PIC = 0.76).

## Discussion

### Efficiency of three microsatellite multiplex panels for the genetic characterization of rice germplasm

Limitations on morphological characterization, including difficulties concerning the definition and validation of neutral traits, experimental costs, evaluation time and genotype × environment interaction are widely discussed in germplasm characterization studies [30,31]. The molecular characterization based on panels of microsatellite markers allowed for an in depth look at the genetic information and organization of the germplasm collection evaluated. The three multiplex panels are the first of a series of panels currently being tested in our laboratory for coverage of all rice chromosomes.

When compared with previous reports by Blair and colleagues [25], who also used multiplex panels for the genotyping of rice cultivars, and by Ni and colleagues [20], who used 111 SSR markers for the evaluation of diversity in rice subspecies, the present study detected a higher average allele number. Garris and colleagues [15] also detected a smaller average allele number than the results presented here for a sample of 234 rice accessions representing the geographic range of *O. sativa*. Such differences in average allele number are probably due to the much smaller number of SSR loci analyzed in this report in comparison to the cited works. Since most loci described here are highly polymorphic regarding the number of different alleles, that is reflected as a higher average allele number for a smaller number of loci. On the other hand, the average PIC value in this study was similar to those reported for *O. sativa *[20,15], and for *japonica *rice accessions [25]. In addition, in comparison with the first two reports, if only *japonica *accessions are considered, our data indicates that values estimated for 485 *japonica *landraces collected in Brazil are much higher. Diversity values such as PIC and *GD*, which account for a more reliable estimate of the value of the SSR markers used, are therefore quite similar to those in previous reports and even higher when only *japonica *accessions are compared. When looking at the analyzed plant material used in the cited studies, one can realize a possible cause for the considerable levels of genetic diversity detected in the present work: never before such a high number of *japonica *accessions of a single country alone had been genotyped and analyzed altogether in multiplex panel studies. Blair and colleagues' work [25] included 27 *japonica *accessions; Ni and colleagues [20]studied 28 *japonica *accessions, while Garris and colleagues [15] analyzed a total of 89 *japonica *accessions.

Microsatellite markers have been used for identification purposes in plants, animals and humans [32-35]. The power of a set of SSR markers for identification of individuals can be measured using different parameters. The matching probability of identical genotypes, also known as the probability of identity (PI), when combined for all loci, represents the likelihood of the presence of two individuals with the same genotype in a population. In other words, it is an estimate of the number of individuals which would have to be analyzed in order to find the same DNA pattern of a randomly selected individual. The combined estimate of 6.4 × 10^-21 ^demonstrates that the probability of finding two accessions of *japonica *rice with the same SSR pattern is almost null when the panels of microsatellite markers discussed here are used. The power of exclusion of the loci composing the three SSR panels is an estimate of the probability of exclusion of a non-parent from a paternity or maternity survey. The combined power of exclusion for the multiplex panels was greater than 99.99%, indicating their ability for parentage determination in rice.

The approach clearly indicates that the use of fluorescently labeled panels of microsatellite markers in semiautomated fashion can greatly contribute to the understanding and management of germplasm collections. The advances in molecular characterization, especially in the possibility of high throughput genotyping of whole collections with a great number of markers distributed throughout the genome open up new opportunities for germplasm characterization. This goal will be achieved if a set of panels based on just one PCR reaction can be developed for the species, such as the three panels described here.

### Upland germplasm diversity and patterns of genetic differentiation

Genetic structure studies have been performed and reported for the two rice cultivated species *O. sativa *and *O. glaberrima *[36,20,15,21,37], but this is the first to focus on traditional landraces collected in Brazil, where rice stands as a major staple food. The plant material used in this study was composed primarily of traditional upland rice cultivars collected in Brazil and kept at the rice germplasm collection of EMBRAPA. These accessions have been collected within a 25-year period in different geographical regions of the country, and only a few have been previously characterized by morphological parameters. The analyzed samples provide a picture of a few hundred years of rice cultivation in the Brazilian territory, where the first introductions of cultivated rice are documented far back to the 16^th ^and 17^th ^centuries [3]. It is believed that the first introduced rice varieties were "red rice" samples from Venice, Italy, brought from Portugal by immigrants from the Azores Islands to the Northern provinces which now comprises the state of Maranhão. As they disseminated among local farmers, these varieties were given the popular names of "Venice rice", "red rice" or "country rice". From there, rice cultivation made its way from the North to the Southern and Mid-Western regions of the country. Production, primarily focusing on *japonica *"red rice" varieties, was later replaced by "white rice" *indica *cultivars.

There is strong evidence that the cluster of 63 accessions would probably have an *indica *genetic background based on both genetic distance and bootstrap analysis. That represents about 11% of the sample of 548 accessions which was referred by preliminary description as composed of *japonica *varieties. Bayesian model-based analysis confirmed the presence of a well defined group containing *indica *accessions. Since there was practically no previous knowledge about the history of the studied germplasm regarding origin, development and introduction in Brazil, the fact that a considerable number of traditional upland rice landraces were mixed with *indica *cultivars is quite intriguing. It is possible that some of these *indica *varieties have been cultivated in lowland production regimes ("varzea"), which differs from the traditional production system of irrigated rice by capitalizing on high watersheds on riverine regions and does not use artificial irrigation. Further investigations would be necessary to more consistently confirm this relationship.

In general, when the different methods applied for clustering and structure analyses were compared – genetic distance and clustering analysis, AMOVA and a Bayesian Model-Based approach – similar patterns of groupings of accessions could be noticed. The distribution of the 475 accessions which shared at least 70% ancestry to one of the three inferred groups is summarized on Table 3. Group 1 consists of 214 accessions, which are colored green on the dendrogram (Additional file 1) and corresponds to a first subcluster of the major *japonica *cluster previously mentioned. These accessions are distributed among all six different origins of collection delimited for the purpose of this analysis (Northern Region, Northeastern Region, Southeastern Region, Southern Region, Mid-Western Region, International accessions), but with a higher prevalence of accessions from the Mid-Western region of Brazil (79%). The second subcluster is represented by Group 2, with 201 accessions, colored blue on the dendrogram, consisting predominantly of accessions collected in Northeastern Brazil (65%), where rice varieties were first introduced in the country. Finally, Group 3 includes 60 accessions plus the control *indica *cultivars IRGA 417 and IRGA 422 CL, corresponding to the minor cluster detected by genetic distance-based neighbor-joining dendrogram construction. Most of them were obtained via exchange of germplasm material with foreign institutions, and are named "International accessions" on Table 3. Accessions belonging to Group 3 are colored red on the dendrogram. Accessions identified as admixed are colored black and are equally distributed among different clusters of the dendrogram.

A search for landrace names provided at the time of the collection trips which might reflect a relation with the oldest rice varieties introduced in Brazil was performed. We found 38 accessions with popular names which might be historically meaningful – those referring to the term "red", as well as those known to be the names of traditional and old rice varieties [3]. Interestingly, 71% of these possessed a higher ancestry coefficient with Group 2. In this group, 51% of all 201 accessions were collected in two neighboring states – Maranhao and Piaui, the region where rice production in Brazil first took place [3]. On the other hand, Group 1 is comprised mostly of accessions collected in the Mid-Western region of the country (54% of all 214 accessions). Inclusion in any of the groups was significantly correlated with accession origin (r = 0.47 p < 0.01). Historically, upland rice production migrated from Maranhao in the North to the Southeastern region and then to the states of Goias and Mato Grosso, both in the Mid-West [3]. Even though we might still be far from a view of the whole scenario, this seems to be an indication of the differentiations which took place on upland rice gene pools as its cultivation in Brazil developed throughout the centuries.

Significant estimates of stratification among the three inferred groups were found, and the strong effects of an autogamous breeding system were detected by Wright's statistics coefficients, as well as Slatkin's coefficient of stratification. Values are consistent with those previously reported [15], where *F*_ST _estimates were 0.20 between tropical and temperate *japonica*, and 0.36 between *indica *and tropical *japonica*. However, when looking at how diversity is partitioned among groups via AMOVA, we noticed that most of the diversity was attributed to differences within groups (88.1%, against 62.5% on Garris' report), rather than among the three inferred groups. A greater partitioning of diversity among, rather than within groups, would be expected for an autogamous species in the absence of human-mediated gene flow. Nevertheless, when compared to a study of maize inbred lines, where 8.3% of the variation was caused by differences among groups [38], our data present comparable values.

Δ*K*, an *ad hoc *quantity related to the second order change of the log probability of data with respect to the number of clusters inferred by the Structure program, proved an useful method for identifying a more probably true value of *K *when there was no previous model or a pre-defined number of groups to rely on. Its rational is to detect the break in slope in the distribution of the log probability values which occurs at the true *K*. In this study, since we expected most of the accessions to belong to the *japonica *subspecies – in addition to the fact that the geographical origins of accessions extended over 22 states in the Brazilian territory – defining a probable number of subgroups prior to an analysis such as the construction of a dendrogram seemed impossible. Our data shows that genetic distance base clustering as well as model-based grouping methods provided consistent results regarding the distribution of accessions among distinct groups (r = 0.867, p < 0.01).

## Conclusion

The data gathered here demonstrates the feasibility of genotyping extensive germplasm collections at marker loci in rice genome using panels of microsatellite markers, each of them multiplexed with a single PCR assay prior to electrophoresis. The accuracy of allele sizing and speed of genotyping would allow the characterization of large collections in short periods of time. The development of algorithms for extracting information from the dataset using straightforward molecular genetic data can possibly expedite improvements in management and use of the germplasm by breeding programs, as well as in the identification, protection and assessments of genetic relationship of rice germplasm. The use of a model-based approach for genetic structure analysis provided important inputs on the knowledge about upland rice germplasm differentiations which happened in Brazil in the last few centuries, since its introduction in the country.

## Methods

### Plant material and DNA extraction

This study included 548 rice (*Oryza sativa *L.) accessions collected mostly in Brazil, but also contained a few accessions from Colombia, the Philippines, Sri Lanka, and other countries, registered in the EMBRAPA germplasm collection (Additional file 2). Two *indica *accessions (IRGA 417 and IRGA 422 CL) were included in the analyses and used as reference for control of allele sizing variation between electrophoresis runs. The two reference accessions are near isogenic lines commercially important in Brazil. The majority of the accessions have been collected in remote rural areas of the country in the last 25 years. The information available for the accessions, although limited, allowed for their initial classification for breeding purposes as *japonica *rice. For most of them, however, none of the available techniques for subspecies classification was ever tested. Knowledge about the genetic background of these accessions was, therefore, an important objective of this study.

Young leaves from five seedlings from each accession were collected for DNA extraction. The plant material was ground inside microcentrifuge tubes using sterile plastic beads by agitation on a Fastprep FP120 (Thermo Savant, Waltham, MA, USA) and the DNA was extracted using a rapid CTAB method as described [39]. DNA concentration was measured in 1% agarose gel after electrophoresis using λ DNA (Invitrogen^®^) as a standard for DNA quantification. DNA was diluted in TE buffer to a final concentration of 3 ng/μL.

### Genotyping using fluorescently-labeled microsatellite panels

Three multiplex panels (A, B and C) consisting of a total of 16 fluorescent-labeled microsatellite loci were used in this study (Table 4). Simultaneous PCR amplifications were carried out in a final volume of 15 μL containing 6 ng of genomic DNA, 0.4 mM of each dNTPs, 0.2 μg/μL BSA, 3 mM MgCl_2_, and 2 U *Taq *DNA Polymerase (Phoneutria^®^, Belo Horizonte, MG, Brazil). For multiplex panel A (5 loci), primer concentrations were 0.2 μM (OS19 and RM248), and 0.13 μM (RM252, RM224 and OG44); for multiplex panel B (6 loci) primer concentrations were 0.13 μM (OG101, OG05 and OG81), 0.2 μM (OG106), 0.23 μM (OG61) and 0.1 μM (RM263); and for panel C (5 loci) primer concentrations were 0.2 μM (RM259), 0.13 μM (RM418 and RM335) and 0.1 μM (RM420 and RM475). Reactions were performed on a GeneAmp PCR System 9700 (Perkin-Elmer, USA) using the following profile: a hot start of 94°C for 5 min, 30 amplification cycles of 1 min at 94°C, 1 min at 52°C (panels A and B)/55°C (panel C), 2 min at 72°C, and a final extension step of 7 min at 72°C. Five microliters of amplification product were combined with 3 μL of loading buffer (98% formamide, 10 mM EDTA, blue dextran) and 2 μL of an internal-lane ROX-labeled size standard [40], followed by denaturation at 95°C for 5 min. An aliquot of 1 μL of the sample was loaded on each lane and run on 4% Longranger polyacrylamide gels in 1× TBE buffer (50-well, 36-cm plates with a 12-cm well-to-read distance), with the recommended run module (constant 30 W) and filter sets C (for panels A and B) and D (for panel C). Gels were run for 2.5 hours on an ABI Prism 377 automatic DNA sequencer (Applied Biosystems^®^, Foster City, CA, USA). Microsatellite fragment sizing was performed using the GeneScan software version 3.1.2 (Applied Biosystems^®^, Foster City, CA, USA). Size standard peaks were user-defined during the analyses. The amplified fragments were assigned as alleles of the appropriate SSR loci using the Genotyper software version 2.5.2 (Applied Biosystems^®^, Foster City, CA, USA). Allele binning was performed by rounding off the Genotyper assigned values to the nearest whole base-pair integer to give a base pair estimate for the allele. Because most of the loci used in this study harbored dinucleotide motifs, the binning process sometimes resulted in intermediate values for the assigned alleles. A correction was performed so that all values would follow the expected size for dinucleotide motif loci, since no previous knowledge about microvariants for the used loci was available. The most frequent allele was considered as a reference for the expected values in this case.

### Statistical analysis

As it was mentioned before, the analyzed accessions had been previously classified as *japonica *rice on its majority. In order to confirm this premise, pairwise genetic distances among the 548 accessions were estimated in order to classify the accessions according to the *indica *or *japonica *genetic background. Genetic distance values were based on the ratio between the sum of the proportions of common alleles between two accessions (Ps) for all loci and twice the number of tested loci [41,42], and were obtained following the parameter [(-ln (Ps)] on the web-based Genetic Distance Calculator [43]. The genetic distance diagonal matrix was submitted to clustering analysis following the Neighbour-Joining method, and a genetic distance dendrogram was built using the NTSYSpc version 2.10z software [44]. In addition, bootstrap analysis of the obtained data was performed so that an estimation of the relative probability of inclusion for any of these accessions in the *japonica *or *indica *subspecies would be obtained. The distribution of allelic frequencies for each subspecies (*indica *and *japonica*) as "baseline populations" was taken as a reference [18]. The relative probability of inclusion was estimated using the Whichrun software [27]. The likelihood that an individual accession may come from one of the source populations (*indica *or *japonica*) is presumed to be equal to the Hardy-Weinberg frequency of its specific genotype at each locus in each respective source population.

Based on the results of the genetic distance and clustering analysis, the accessions classified as *japonica *rice were used to evaluate the performance of the three marker panels in comparison to previously reported multiplex marker analyses using the program PowerMarker v.3.23 [45]. Estimates of allele number, observed heterozygosity (Ho), gene diversity under Hardy-Weinberg equilibrium (HWE) and polymorphism information content (PIC) were calculated. Fisher's exact test was applied to individual marker loci to test the conformity to HWE expectations. Expected gene diversity was calculated based on the unbiased estimator formed by multiplying the sample expected heterozygosity (1 - Σ_*i *_*p*_*i*_^2^) by the factor (2*n*)/(2*n *- 1); being *p*_*i *_the frequency of the *i*th allele for each locus and *n *the number of analyzed samples [47]. A database of allelic frequencies for all loci was established using PowerMarker v.3.23 [45]. The combined efficiency of the panels for questions regarding line discrimination, seed contamination or hybrid origin (paternity analysis) was estimated by parameters such as matching probability and power of exclusion (PE). The matching probability or the probability of identical genotypes [48], defined as PI = Σ*p*_*i*_^4 ^+ Σ(2*p*_*i*_*p*_*j*_)^2^, was estimated for the selected loci individually, and later, for all loci at once. The power of exclusion, the probability of excluding a random individual from the population as a potential parent of an offspring based on the genotype of one parent and offspring, was calculated as PE = Σ*p*_*i *_(1-*p*_*i*_)^2 ^- 1/2 Σ*p*_*i*_^2^*p*_*j*_^2 ^[49].

The genetic structure of the germplasm collection was analyzed according to a contrast between an *a priori *model of population structure based on the clusters defined by the genetic distance analysis and an unknown *a priori *model using the software Structure version 2.1 [50,28]. Genetic distance and cluster analysis were initially used as a reference to depict possible signs of structuring, suggesting potential composition of subpopulations. For comparison purposes, the analyses were performed both on the complete set of 548 accessions and on the set of 485 *japonica *accessions using a burn-in period of 20,000 in the model-based program Structure, followed by a run length of 200,000. Five independent runs for each *K *– the number of inferred groups estimated by Structure – were performed, with *K *values ranging from 1 to 15. The model choice criterion to detect the most probable value of *K *was Δ*K*, an *ad hoc *quantity related to the second order change of the log probability of data with respect to the number of clusters inferred by Structure [29]. An accession was included in a particular cluster inferred by the program if at least 70% of its genome value, as measured by its membership coefficient (ranging from 0 to 1), was estimated to belong to that cluster. Overall *F*_ST _values for the inferred clusters were calculated using PowerMarker. The correlation between clusters defined by Structure and clusters defined by genetic distance analysis followed by Neighbor-Joining grouping was estimated by Pearson's correlation coefficient.

The extent of genetic differentiation among groups, as defined *a priori *by the genetic distance and clustering analysis, was also estimated under the premises of the infinite allele model (*F*_ST_) [51] and under the stepwise mutation model (*R*_ST_) [52]. Analysis of molecular variance (AMOVA) was also employed to evaluate the substructuring level of the collection using the program PowerMarker.

The majority of the accessions (Additional file 2) have been collected in five major geographic regions of Brazil (Northern Region, Northeastern Region, Southeastern Region, Southern Region, Mid-Western Region) and a few originated in other countries (International accessions). The correlation between geographic origin and *F*_ST _values of the collection was analyzed by Pearson's correlation coefficient.

## Authors' contributions

MPF designed and optimized multiplex panel C, performed genotyping of accessions with the three panels, as well as statistical analyses and drafting of the manuscript. AB designed and optimized multiplex panels A and B, and assisted in drafting the manuscript. AANA performed genotyping of accessions with the three panels, PHNR selected and provided the plant material used in this study. MEF conceived and supervised the study, performed some statistical analysis and edited the manuscript. All authors read and approved the final manuscript.

## Supplementary Material

Additional File 1 Neighbor-joining dendrogram based on pairwise genetic distances for 548 rice accessions genotyped with 16 SSR markers. The different colors refer to the inferred clusters from the Structure program. Green – Group 1; Blue – Group 2; Red – Group 3; Black – Admixed Click here for file

Additional File 2 Rice accessions belonging to the EMBRAPA germplasm bank analyzed in this study Click here for file

## Figures and Tables

**Table 1:** Total number of observations for all 485 genotyped accessions, number of unique alleles for each marker, gene diversity (*GD*), observed heterozygosity (*H*) and PIC values

**Marker**	**Allele no.**	***GD***	***H***	**PIC**
**RM248**	11	0.6753	0.0451	0.6219
**OG44**	20	0.8192	0.0167	0.7949
**RM224**	12	0.7457	0.0240	0.7135
**RM252**	14	0.6680	0.0795	0.6265
**OS19**	12	0.6646	0.0326	0.6410
**OG81**	12	0.7777	0.0803	0.7438
**RM263**	15	0.8056	0.0695	0.7802
**OG61**	24	0.8478	0.0706	0.8327
**OG05**	13	0.5874	0.0355	0.5471
**OG101**	19	0.7130	0.0275	0.6899
**OG106**	26	0.9197	0.0293	0.9145
**RM335**	14	0.7967	0.0359	0.7696
**RM420**	8	0.4973	0.0205	0.4165
**RM418**	8	0.3818	0.0204	0.3602
**RM259**	12	0.7324	0.0108	0.7048
**RM475**	9	0.0414	0.0000	0.0413

**Average**	14	0.6671	0.0374	0.6374

**TOTAL**	229	-	-	-

**Table 2:** Comparisons among Bayesian inferred groups regarding genetic diversity estimates and group differentiation

	**Diversity**				***F***_ST_		
		
**Inferred group**	**n**	**Allele No.**	***GD***	***H***	**1**	**2**	**Overall**
**1**	214	9	0.539	0.028	-		
**2**	201	12	0.681	0.047	0.17	-	
**3**	62	10	0.793	0.063	0.31	0.18	0.177

**Table 3:** Model-based partitioning of ancestry of 476 accessions of *O. sativa *spp. *japonica *using SSR-marker data

	**Inferred Groups**			
		
**Origin**	**1**	**2**	3*	**No. of accessions**
Northern Region	0.59	0.36	0.05	39
Northeastern Region	0.19	0.65	0.16	172
Southeastern Region	0.5	0.31	0.19	68
Southern Region	0.31	0.54	0.15	26
Mid-Western Region	0.79	0.18	0.03	146
International Accessions	0.08	0.5	0.42	24

*Group 3 represents accessions with an *indica *background

**Table 4:** Fluorescently-labeled microsatellite markers which compose the three multiplex panels (Panel A, Panel B and Panel C)

Panel	Loci	Fluorescent Dye	Color	Expected Size range	Chrom.	Motif	Reference
A	RM252	**TET**	**Green**	**194–274**	**4q**	**(ag)n**	**[53]**
A	RM224	**TET**	**Green**	**124–162**	**11q**	**(aag)_8_(ag)_13_g(ag)**	**[53]**
A	OG44	**6-FAM**	**Blue**	**152–172**	**3q**	**(ct)_4_-23pb-(ct)_22_(gt)_4_(gc)_6_**	**[8]**
A	RM248	**6-FAM**	**Blue**	**72–108**	**7q**	**(ag)n**	**[53]**
A	OS19	**HEX**	**Yellow**	**174–198**	**6p**	**(ag)n**	**[54]**
							
B	OG101	**TET**	**Green**	**95–161**	**2p**	**(ag)n**	**[8]**
B	OG106	**TET**	**Green**	**178–254**	**9p**	**(ag)n**	**[8]**
B	OG81	**6-FAM**	**Blue**	**71–89**	**1**	**(ag)n**	**[8]**
B	RM263	**6-FAM**	**Blue**	**147–195**	**2q**	**(ag)n**	**[53]**
B	OG05	**HEX**	**Yellow**	**170–194**	**3p**	**(ag)n**	**[8]**
B	OG61	**HEX**	**Yellow**	**96–154**	**5q**	**(ag)n**	**[8]**
							
C	RM335	**6-FAM**	**Blue**	**104–156**	**4p**	**(ctt)**_25_	**[55]**
C	RM420	**6-FAM**	**Blue**	**183–201**	**7q**	**(aaat)**_7_	**[7]**
C	RM418	**6-FAM**	**Blue**	**253–311**	**7q**	**(att)**_21_	**[7]**
C	RM259	**NED**	**Yellow**	**155–176**	**1p**	**(ct)**_17_	**[53]**
C	RM475	**NED**	**Yellow**	**186–246**	**2q**	**(tatc)**_8_	**[7]**
