# Supplementary figures and images for: A set of multiplex panels of microsatellite markers for rapid molecular characterization of rice accessions
Source: BMC Plant Biol. 2007 May 21;7:23. doi: 10.1186/1471-2229-7-23 (PMC1888689; doi:10.1186/1471-2229-7-23)

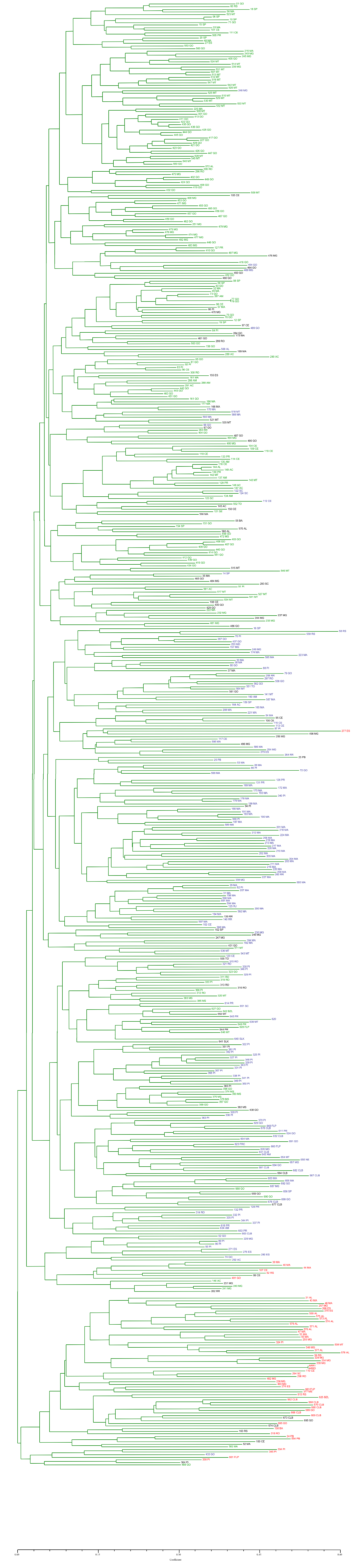

Supplement: Additional File 1 — Neighbor-joining dendrogram based on pairwise genetic distances for 548 rice accessions genotyped with 16 SSR markers. The different colors refer to the inferred clusters from the Structure program. Green – Group 1; Blue – Group 2; Red – Group 3; Black – Admixed [file 1471-2229-7-23-S1.png]
