# Supplementary material for: A set of multiplex panels of microsatellite markers for rapid molecular characterization of rice accessions
Source: BMC Plant Biol. 2007 May 21;7:23. doi: 10.1186/1471-2229-7-23 (PMC1888689; doi:10.1186/1471-2229-7-23)
Supplement: Additional File 2 — Rice accessions belonging to the EMBRAPA germplasm bank analyzed in this study [file 1471-2229-7-23-S2.doc]

S1 – Rice accessions belonging to the EMBRAPA germplasm bank analyzed in this study.

| **Sample ID** | **Accession code** | **Accession name** | **Year of collection** | **Collection site*** |
| --- | --- | --- | --- | --- |
| 01_GO | CA780003 | LEVANTA HOMEM | 1983 | GO |
| 02_GO | CA780005 | PRINCESA | 1983 | GO |
| 05_BA | CA780013 | MARANHAO VERMELHO | 1983 | BA |
| 06_SP | CA780019 | SEQUEIRO OU PARANA | 1983 | SP |
| 08_SP | CA780024 | CORTE | 1983 | SP |
| 09_SP | CA780026 | ESTANCIA BOA VISTA | 1983 | SP |
| 10_SP | CA780030 | ITAINHAIM | 1983 | SP |
| 12_SP | CA780033 | ENCHE TULHA | 1983 | SP |
| 14_SP | CA780040 | PRECOCE FERRAO PRETO | 1983 | SP |
| 15_SP | CA780043 | BAMBU OU GUAPA | 1983 | SP |
| 16_SP | CA780044 | CATETO SEDA | 1983 | SP |
| 18_SP | CA780048 | HIBRIDO 4 MESES | 1983 | SP |
| 19_SP | CA780051 | CATETO BICO PRETO | 1983 | SP |
| 20_SP | CA780053 | AMARELAO PRECOCE | 1983 | SP |
| 21_AL | CA780055 | ESAV-36 | 1994 | AL |
| 23_PB | CA780059 | MARUIM | 1983 | PB |
| 24_PB | CA780061 | TRES POTES | 1983 | PB |
| 25_PB | CA780064 | SACOREMA | 1983 | PB |
| 27_ES | CA780080 | KAKI OU AMARELAO | 2000 | ES |
| 28_MA | CA780090 | MANGABA | 1984 | MA |
| 29_MA | CA780096 | LISAO | 1994 | MA |
| 30_MA | CA780097 | CUTIAO VERMELHO | 2000 | MA |
| 31_MA | CA780098 | NENE VERMELHO | 1984 | MA |
| 32_MA | CA780100 | TRES MESES BRANCO | 1983 | MA |
| 33_MA | CA780115 | RIO BRANCO | 1983 | MA |
| 35_MA | CA780117 | CEM DIAS | 1983 | MA |
| 37_MA | CA780123 | GUATAMBU | 1983 | MA |
| 38_MA | CA780126 | OURO | 1983 | MA |
| 39_MA | CA780127 | VENEZ ROXO | 1983 | MA |
| 40_MA | CA780128 | TAQUARAO | 1983 | MA |
| 43_MA | CA780136 | VASSOURINHA | 1983 | MA |
| 44_MA | CA780139 | CATARINA | 1983 | MA |
| 46_MA | CA780141 | CANELA ROXA | 1983 | MA |
| 47_MA | CA780143 | PIRRACA | 1983 | MA |
| 48_MA | CA780148 | BRANCO PRECOCE | 1983 | MA |
| 49_MA | CA780151 | STO ANTONIO | 1983 | MA |
| 51_MA | CA780157 | ARROZ DE DEUS | 1983 | MA |
| 52_MA | CA780158 | CAJUEIRO LISO | 1983 | MA |
| 53_MA | CA780162 | MORRO DA GARCA | 1983 | MA |
| 54_MA | CA780164 | COQUINHO | 1983 | MA |
| 55_MA | CA780166 | MULATINHO | 1983 | MA |
| 56_MA | CA780167 | SEMENTE DE PAIOL | 1983 | MA |
| 57_MA | CA780168 | 3 MESES BRANCO COMPRIDO | 1983 | MA |
| 58_RS | CA780170 | FORMOSA | 1983 | RS |
| 59_RS | CA780171 | CACHINHO | 1983 | RS |
| 60_RS | CA780174 | BICO TORTO | 1983 | RS |
| 62_RS | CA780180 | CACHIMBO DO SECO | 1983 | RS |
| 63_GO | CA780183 | AMARELO BICO GANGA | 1983 | GO |
| 65_GO | CA780185 | BARRANQUEIRA | 1983 | GO |
| 66_GO | CA780188 | CARIOQUINHA VERMELHO | 1983 | GO |
| 67_GO | CA780189 | CARIOQUINHA AREIA | 1983 | GO |
| 70_PI | CA780199 | GRAO DE OURO | 1983 | PI |
| 71_GO | CA780201 | GEM OU BRASIL | 1983 | GO |
| 72_GO | CA780202 | ARROZ MARANHAO | 1983 | GO |
| 73_GO | CA780204 | RENDIMENTO | 1983 | GO |
| 74_GO | CA780206 | GAIPA | 1983 | GO |
| 75_GO | CA780207 | CARIOCAO | 1983 | GO |
| 76_GO | CA780210 | CANTA GALO | 1983 | GO |
| 77_GO | CA780211 | FERNANDO | 1983 | GO |
| 78_GO | CA780215 | AGULHINHA MARANHAO | 1983 | GO |
| 79_GO | CA780217 | PUTECA | 1983 | GO |
| 80_GO | CA780219 | FERRINHO | 1983 | GO |
| 81_GO | CA780220 | CARIJO | 1983 | GO |
| 82_PI | CA780222 | PARAMBU | 1983 | PI |
| 83_PI | CA780226 | RONDON | 1983 | PI |
| 84_PI | CA780228 | MEIO AGULHA | 1983 | PI |
| 85_PI | CA780230 | MACABA (LIGEIRO) | 1983 | PI |
| 86_PI | CA780232 | GERGELINHO | 1983 | PI |
| 87_PI | CA780234 | VERMELHO TALO ROXO | 1983 | PI |
| 88_PI | CA780236 | AGULHA VERMELHO | 1983 | PI |
| 89_PI | CA780237 | SAQUAREMA ROXO | 1983 | PI |
| 90_PI | CA780238 | PEDREGULHO | 1983 | PI |
| 91_PI | CA780239 | 101 CATALAO | 1983 | PI |
| 92_PI | CA780245 | CUTIAO LIGEIRO | 1983 | PI |
| 93_PI | CA780249 | CAROCO-SO | 1983 | PI |
| 94_PI | CA780258 | MACABA (TARDIO) | 1983 | PI |
| 95_CE | CA780261 | PRATA BRANCO | 1983 | CE |
| 96_CE | CA780262 | AGULHA DO ESTADO | 1983 | CE |
| 97_CE | CA780264 | IGUAPE DOURADO | 1983 | CE |
| 98_CE | CA780265 | GUAPE VERDADEIRO | 1983 | CE |
| 99_CE | CA780266 | GAVIAO | 1983 | CE |
| 100_CE | CA780269 | PIOJOTA | 1983 | CE |
| 101_CE | CA780270 | AGULHA PRETA | 1983 | CE |
| 102_CE | CA780273 | PRATA LIGEIRO | 1983 | CE |
| 104_CE | CA780275 | GUAPE (DOURADO LIGEIRO) | 1983 | CE |
| 105_CE | CA780279 | CANO ROXO (LIGEIRO) | 1983 | CE |
| 106_CE | CA780281 | CANO ROXO | 1983 | CE |
| 107_CE | CA780283 | AGULHA DOURADO | 1983 | CE |
| 108_CE | CA780284 | JAVANES | 1983 | CE |
| 109_CE | CA780287 | PAULISTA DOURADO | 1983 | CE |
| 110_CE | CA780288 | MORUIM-AGULHA | 1983 | CE |
| 111_CE | CA780294 | MERUIM DE TALO ROXO | 1983 | CE |
| 112_CE | CA780295 | SANTO AMERICO | 1983 | CE |
| 113_CE | CA780299 | MERUIM LIGEIRO | 1985 | CE |
| 114_CE | CA780300 | ARCOS BRANCO | 1983 | CE |
| 115_CE | CA780301 | MARANHAO (VERDADEIRO) | 1983 | CE |
| 116_CE | CA780303 | LIMEIRA | 1983 | CE |
| 117_CE | CA780305 | NENEN CANA ROXA | 1983 | CE |
| 118_CE | CA780306 | DOURADINHO LIGEIRO | 1983 | CE |
| 119_CE | CA780308 | AMARELAO LIGEIRO | 1983 | CE |
| 120_CE | CA780309 | ARROZ DE GUERRA | 1983 | CE |
| 121_SE | CA780311 | CHATINHO BRANCO | 1983 | SE |
| 122_SC | CA780314 | PRAINO | 1983 | SC |
| 123_SC | CA780316 | NOVA CHINA | 1983 | SC |
| 124_SC | CA780317 | PARANA | 1983 | SC |
| 125_RJ | CA780322 | PONTA ROXA | 1983 | RJ |
| 126_PR | CA780324 | CACHO DUPLO | 1983 | PR |
| 127_PR | CA780327 | SEMENTE COMUM | 1983 | PR |
| 128_PR | CA780329 | COMUM CREOLO | 1983 | PR |
| 129_PR | CA780333 | CAMPINEIRO | 1983 | PR |
| 130_PR | CA780334 | STA AMELIA | 1983 | PR |
| 131_PR | CA780336 | PRATAO GOIANO | 1985 | PR |
| 132_PR | CA780338 | AMARELAO TARDIO | 1983 | PR |
| 133_PR | CA780340 | AMARELINHO 4 MESES | 1983 | PR |
| 134_AM | CA780343 | AGULHAO | 1996 | AM |
| 135_AM | CA780344 | BICUDO | 1985 | AM |
| 137_AM | CA780347 | 6 MESES | 2000 | AM |
| 139_RR | CA780352 | AGULHA BRANCO RR | 1996 | RR |
| 140_RR | CA780354 | ZEBU(PINGO D'AGUA) | 2000 | RR |
| 141_MT | CA780357 | ARACATUBA | 1983 | MT |
| 142_MT | CA780358 | ARROZ | 1983 | MT |
| 143_MT | CA780359 | SANTA AMERICA | 2000 | MT |
| 144_AL | CA780362 | MNEIRO | 1996 | AL |
| 145_AC | CA780365 | DOURADINHO | 1994 | AC |
| 146_AC | CA780368 | MACAPA | 2000 | AC |
| 147_AC | CA780369 | ARROZ MINEIRO | 1996 | AC |
| 148_AC | CA780373 | ARROZ TEIMOSO | 2000 | AC |
| 149_AC | CA780374 | AGULHA BRANCO | 2000 | AC |
| 150_CE | CA780378 | IGUAPE REDONDO | 1983 | CE |
| 151_GO | CA780380 | MIMOSO | 1984 | GO |
| 152_SP | CA780383 | CEDINHA | 1998 | SP |
| 154_SP | CA780389 | PRECOCE AMARELO | 1997 | SP |
| 155_ES | CA780390 | MINEIRINHO | 2000 | ES |
| 156_GO | CA780393 | BEIRA MAR | 1984 | GO |
| 157_MG | CA780395 | PRATA ROXA | 1984 | MG |
| 158_BA | CA780397 | SAQUAREMA | 1994 | BA |
| 159_SP | CA780400 | BATATAIS COIMBRA | 1984 | SP |
| 160_AM | CA780401 | AGULHINHA CAQUI | 1984 | AM |
| 161_GO | CA780402 | ARROZ DE 64 DIAS | 1995 | GO |
| 163_RS | CA780409 | ITAQUI | 1994 | RS |
| 164_AC | CA780410 | MERUIM | 2000 | AC |
| 165_MA | CA780412 | COMUM BRANCO | 2000 | MA |
| 168_MA | CA790013 | POUPA PREGUICA | 2000 | MA |
| 169_MA | CA790017 | MEARIM VERMELHO | 1997 | MA |
| 170_MA | CA790018 | MARABA | 1997 | MA |
| 172_MA | CA790021 | MEARIM MIUDO | 1997 | MA |
| 173_MA | CA790032 | PALHA MURCHA | 1997 | MA |
| 174_MA | CA790040 | VERMELHO DO MARANHAO | 1997 | MA |
| 175_MA | CA790042 | RABO DE BURRO | 1997 | MA |
| 177_MA | CA790048 | CUTIÃO BRANCO | 1997 | MA |
| 178_MA | CA790051 | ARROZ CASADO | 2000 | MA |
| 179_MA | CA790052 | LIGEIRO VERMELHO | 1997 | MA |
| 180_MA | CA790054 | DOBRADINHO | 1997 | MA |
| 181_MA | CA790067 | ARROZ DE LEITE | 1997 | MA |
| 183_MA | CA790076 | BACABA | 1997 | MA |
| 184_MA | CA790079 | GOIANO | 1997 | MA |
| 186_MA | CA790094 | CUCHILAO | 2000 | MA |
| 187_MA | CA790112 | POUPA PREGUICA OU MARABA | 2000 | MA |
| 189_MA | CA790138 | SAGRIMAO | 1997 | MA |
| 190_MA | CA790157 | BACABINHA | 1983 | MA |
| 191_MA | CA790163 | VERDADEIRO | 1983 | MA |
| 192_MA | CA790176 | BURITI VERMELHO | 2000 | MA |
| 193_MA | CA790178 | TALO ROXO | 2000 | MA |
| 194_MA | CA790181 | PINDARE | 1983 | MA |
| 198_MA | CA790207 | CANELA DE ACO | 2000 | MA |
| 199_MA | CA790211 | MURUIM VERMELHO | 2000 | MA |
| 200_MA | CA790220 | PELA MAO | 1983 | MA |
| 201_MA | CA790231 | CANA ROXA BRANCO | 2000 | MA |
| 202_MA | CA790236 | TALO ROXO (BACABINHO) | 2000 | MA |
| 203_MA | CA790238 | JATOBA | 2000 | MA |
| 204_MA | CA790241 | MURUIM BRANCO | 1983 | MA |
| 205_MA | CA790248 | TORO | 1983 | MA |
| 206_MA | CA790256 | COME CRU BRANCO | 2000 | MA |
| 207_MA | CA790266 | NENEZINHO BRANCO | 1983 | MA |
| 208_MA | CA790268 | ARROZ COMUM | 2000 | MA |
| 209_MA | CA790282 | CHATAO VERMELHO | 2000 | MA |
| 210_MA | CA790293 | CANA ROXA VERMELHO | 2000 | MA |
| 211_MA | CA790301 | LIGEIRO | 2000 | MA |
| 212_MA | CA790308 | SACAREMA | 2000 | MA |
| 213_MA | CA790311 | PIAUI | 2000 | MA |
| 215_MA | CA790325 | GUAIRA BRANCO | 2000 | MA |
| 216_MA | CA790328 | VERMELHO | 2000 | MA |
| 217_MA | CA790334 | MIUDO BRANCO | 2000 | MA |
| 218_MA | CA790337 | MURUIM BRANCO | 2000 | MA |
| 219_MA | CA790340 | BACABA BRANCO | 2000 | MA |
| 220_MA | CA790346 | NENEN | 2000 | MA |
| 221_MA | CA790347 | RABO DE BURRO | 2000 | MA |
| 222_MA | CA790349 | CHATAO CANA ROXA | 2000 | MA |
| 223_MA | CA790359 | TORÓ VERMELHO | 2000 | MA |
| 224_MA | CA790360 | ZEBU BRANCO | 2000 | MA |
| 225_MA | CA790363 | COME CRU VERMELHO | 1995 | MA |
| 226_MA | CA790365 | COME CRU | 2000 | MA |
| 227_MA | CA790397 | TALO ROXO (BACABINHO) | 2000 | MA |
| 228_MG | CA800001 | SANTA CATARINA | 2000 | MG |
| 229_MG | CA800015 | DOURADAO | 1984 | MG |
| 230_MG | CA800015A | MACARICO | 1994 | MG |
| 231_MG | CA800020 | DOURADAO/AMARELAO | 1984 | MG |
| 232_MG | CA800026 | MANGOTE | 2000 | MG |
| 233_MG | CA800034 | IGUAPE SEM ARESTA | 1984 | MG |
| 234_MG | CA800041 | AMARELINHO/PAGA DIVIDA | 1994 | MG |
| 235_MG | CA800049 | MUNDICERA | 2000 | MG |
| 236_MG | CA800050 | VENES BRANCO | 1994 | MG |
| 237_MG | CA800068 | AMARELAO/DOURADAO | 2000 | MG |
| 238_MG | CA800078 | CACHO DE OURO | 2000 | MG |
| 239_MG | CA800080 | UBERABINHA | 1984 | MG |
| 240_MG | CA800081 | BREJEIRO/NENENZINHO | 1984 | MG |
| 241_MG | CA800082 | BREJEIRO | 2000 | MG |
| 243_MG | CA800084 | CANA ROXA(LISO) | 1984 | MG |
| 244_MG | CA800091 | QUEBRA CACHO | 1984 | MG |
| 245_MG | CA800093 | BICO DE CURIO | 1984 | MG |
| 246_MG | CA800101 | SERRA AZUL | 1984 | MG |
| 247_MG | CA800102 | CANA ROXA C/PALHA AMARELA | 1984 | MG |
| 248_MG | CA800103 | CHORINHO | 2000 | MG |
| 249_MG | CA800108 | CHORINHO AMERICANO | 1994 | MG |
| 251_MG | CA800111 | COQUEIRO CASCA BRANCA | 1995 | MG |
| 253_MG | CA800118 | CHORINHO C/ APICULO ESCURO | 2000 | MG |
| 254_MG | CA800123 | PRATA | 1984 | MG |
| 255_MG | CA800124 | NANICO | 1994 | MG |
| 256_MG | CA800127 | BICO DE ROLA | 1984 | MG |
| 257_MG | CA800128 | BARRIGA BRANCA | 1994 | MG |
| 258_RR | CA800143 | AGULHINHA TARDIO | 1984 | RR |
| 260_RR | CA800150 | VERMELHAO | 1984 | RR |
| 262_RR | CA800154 | AGULHAO BRANCO | 1997 | RR |
| 263_RR | CA800171 | COMUM CHATAO | 1984 | RR |
| 264_RR | CA800178 | ARROZ AGULHINHA | 1997 | RR |
| 267_GO | CA800192 | ARROZ CARIOQUINHA | 1994 | GO |
| 268_ES | CA810003 | HIBRIDO | 1997 | ES |
| 270_ES | CA810016 | DOIDAO | 1997 | ES |
| 271_ES | CA810017 | ARROZ DE MORRO | 2000 | ES |
| 274_ES | CA810030 | JAGUARAO OU TAQUARAO | 2000 | ES |
| 276_ES | CA810038 | SAMABAIA | 1997 | ES |
| 277_ES | CA810039 | ARROZ BARRIGA BRANCA | 1997 | ES |
| 279_ES | CA810049 | SAMAMBAIA BRANCO | 2000 | ES |
| 280_ES | CA810050 | SAMABAIA AMARELO | 1997 | ES |
| 284_GO | CA810069 | ARROZ 4 MESES | 2000 | GO |
| 286_AM | CA820035 | AMARELAO BICO PRETO | 1997 | AM |
| 287_AM | CA820040 | JAGUARIZINHO | 1997 | AM |
| 288_AM | CA820047 | ARROZ MONTANHA | 1998 | AM |
| 289_AC | CA820058 | AGULHAO PAULISTA | 1998 | AC |
| 290_AC | CA820069 | PAULISTA | 1997 | AC |
| 291_AC | CA820071 | ARROZ CANHOTO | 1997 | AC |
| 292_AC | CA820096 | ARROZ BOLIVIANO/ARROZ 25 | 1983 | AC |
| 293_SC | CA820103 | ARROZ CAROLINO | 1998 | SC |
| 294_SC | CA830003 | ARROZ AMARELO E BRANCO | 1984 | SC |
| 295_RO | CA830005 | PRATAO DE SAO PAULO | 1984 | RO |
| 296_RO | CA830027 | A.AGULHADO/P.MINEIRO | 1984 | RO |
| 297_RO | CA830028 | AGULHINHA AMARELO | 1998 | RO |
| 298_RO | CA830029 | AGULHINHA BRANCO | 1998 | RO |
| 299_RO | CA830032 | A.BOLINHA/CATETINHO | 1984 | RO |
| 300_RO | CA830034 | JAPONES BRANCO | 1984 | RO |
| 311_RO | CA830087 | ARROZ BAMBU | 1984 | RO |
| 312_RO | CA830088 | ARROZ OURO VERDE | 1984 | RO |
| 313_RO | CA830095 | ARROZ AMARELAO | 2000 | RO |
| 314_RO | CA830111 | TIBIRINHA | 1984 | RO |
| 315_RO | CA830113 | TAQUARAMA | 1984 | RO |
| 316_RO | CA830117 | BICO GANGA BRANCO | 1984 | RO |
| 318_RO | CA830125 | CANELA CURTA/PIRANAO | 1984 | RO |
| 319_RO | CA830128 | MONTANHA | 1984 | RO |
| 320_MT | CA830130 | AGULHINHA DO PARAGUAI | 1984 | MT |
| 321_RO | CA830132 | PRATAO 5 MESES | 1984 | RO |
| 322_PI | CA830133 | ARROZ CATETAO | 1984 | PI |
| 323_GO | CA840002 | ARROZ ROXO | 1985 | GO |
| 324_PI | CA840022 | MACABA MIUDO | 1985 | PI |
| 325_PI | CA840023 | GERGELIM | 1998 | PI |
| 327_PI | CA840025 | MUCUIM | 1985 | PI |
| 328_PI | CA840026 | ARROZ COCO | 1998 | PI |
| 329_PI | CA840027 | MURUIM | 2000 | PI |
| 330_PI | CA840028 | ARROZ CLASSIFICADO | 1998 | PI |
| 331_PI | CA840030 | MARANHENSE | 1996 | PI |
| 332_PI | CA840035 | ARROZ VERMELHO / LIGEIRO | 1997 | PI |
| 333_PI | CA840036 | ESPETO DE FERRO | 1985 | PI |
| 334_PI | CA840042 | LAGEADO LEGITIMO | 1996 | PI |
| 335_PI | CA840044 | LIGEIRO DOURADO | 1998 | PI |
| 336_PI | CA840045 | ARROZ ASA | 1996 | PI |
| 337_PI | CA840049 | ARROZ DO SUL | 1996 | PI |
| 338_PI | CA840055 | LIGEIRO BRANCO | 1985 | PI |
| 339_PI | CA840056 | LIGEIRO PINTADO | 1996 | PI |
| 340_PI | CA840058 | ARROZ CABELUDO | 1998 | PI |
| 341_PI | CA840059 | LIGEIRO/ENCHE QUARTA | 1985 | PI |
| 342_PI | CA840060 | ENCHE QUARTA/TARDAO | 1985 | PI |
| 343_PI | CA840067 | ZEBU LIGEIRO | 2000 | PI |
| 344_PI | CA840068 | VERMELHINHO | 1998 | PI |
| 345_PI | CA840069 | LIGEIRINHO DESCONHECIDO | 1985 | PI |
| 346_PI | CA840082 | ENCHE PAIOL | 1998 | PI |
| 348_PI | CA840087 | MOCO | 1998 | PI |
| 349_PI | CA840089 | PINGO DE OURO | 2000 | PI |
| 350_PI | CA840090 | MARANHAO PRETO | 1985 | PI |
| 351_PI | CA840098 | MATO GROSSO | 1998 | PI |
| 357_PI | CA840147 | CASADO LIGEIRO | 1999 | PI |
| 358_PI | CA840148 | FUNDO ROXO | 1999 | PI |
| 361_PI | CA840152 | SAO PAULO BRANCO | 1999 | PI |
| 363_PI | CA840154 | MIUDO ROXO | 1999 | PI |
| 364_PI | CA840155 | FARTURA | 1999 | PI |
| 365_PI | CA840161 | ENCHE QUARTO | 1999 | PI |
| 366_PI | CA840167 | BAIANO | 1999 | PI |
| 368_PI | CA840174 | GUIBRA | 1997 | PI |
| 369_PI | CA840176 | MARANHENCE LIGEIRO | 2000 | PI |
| 370_PI | CA840179 | MARANHAOZINHO | 1999 | PI |
| 374_MS | CA850005 | ARROZ MIUDO | 2000 | MS |
| 375_MS | CA850018 | 4 MESES BRANCO | 2000 | MS |
| 377_MS | CA850023 | ARROZ DE MAIO | 2000 | MS |
| 379_MS | CA850028 | ARROZ DOS INDIOS | 2000 | MS |
| 382_MS | CA850041 | CAROLINA/BICO PRETO | 2000 | MS |
| 383_MS | CA850048 | ARROZ AMARELINHO | 2000 | MS |
| 385_MS | CA850053 | AMARELO 5 MESES | 2000 | MS |
| 393_MS | CA850078 | CAROLINA LONGO | 2000 | MS |
| 397_GO | CA860014 | BRANCO DE ANICUNS | 2000 | GO |
| 398_GO | CA860019 | 5 MESES BRANCO | 2000 | GO |
| 399_GO | CA860022 | ARROZ DE AMERICANO | 1999 | GO |
| 401_GO | CA860043 | BICO BRANCO 3 MESES | 2000 | GO |
| 402_GO | CA860044 | BICO MARROM | 2000 | GO |
| 403_GO | CA860049 | ARROZ MEIO AGULHA | 2000 | GO |
| 404_GO | CA860055 | BICO PRETO DA CANA ROXA | 1999 | GO |
| 405_GO | CA860063 | GUAIRA DE 3 MESES | 1999 | GO |
| 406_GO | CA860067 | BICO GANGA IANO | 2000 | GO |
| 407_GO | CA860068 | BICO ROXO DA CANA ROXA | 2000 | GO |
| 408_GO | CA860069 | 4 MESES BICO BRANCO | 1999 | GO |
| 409_GO | CA860070 | BICO PRETO CASCA BRANCA | 2000 | GO |
| 410_GO | CA860073 | ALVORADA | 2000 | GO |
| 411_GO | CA860074 | ARROZ AMARELO/BRANCO | 2000 | GO |
| 412_GO | CA860083 | IGUAPE | 1999 | GO |
| 413_GO | CA860085 | ARROZ ALVORADA | 2000 | GO |
| 414_GO | CA860087 | BICO PRETO DE CARMO | 1999 | GO |
| 415_GO | CA860088 | ARROZ RENDOSO | 2000 | GO |
| 416_GO | CA860089 | ARROZ BRANCO BICO PRETO | 2000 | GO |
| 417_GO | CA860102 | COMUM | 2000 | GO |
| 419_GO | CA860105 | ARROZ 3 MESES ANTIGO | 1999 | GO |
| 421_GO | CA860111 | IGUAPAO | 1999 | GO |
| 422_GO | CA860114 | 3 MESES/4 MESES | 1999 | GO |
| 423_GO | CA860115 | ARROZ SEMPRE VERDE | 1999 | GO |
| 424_GO | CA860116 | BICO GANGA AMARELO | 1999 | GO |
| 425_GO | CA860117 | 3 MESES AMARELO/CEM DIAS | 1999 | GO |
| 426_GO | CA860118 | ARROZ RUBIATABA | 2000 | GO |
| 427_GO | CA860124 | BICO ROXO BRANCO | 1999 | GO |
| 428_GO | CA860126 | BRANCO DE ITAPACI | 2000 | GO |
| 429_GO | CA860127 | BICO GANGA CURTO | 1999 | GO |
| 430_GO | CA860134 | BICO PRETO COM ARISTA | 1999 | GO |
| 431_GO | CA860137 | ARROZ PRATINHA | 2000 | GO |
| 432_GO | CA870004 | 4 MESES AMARELO | 1996 | GO |
| 433_GO | CA870007 | ARROZ PELUDO | 1996 | GO |
| 434_GO | CA870008 | PRATINHA BRANCO | 1999 | GO |
| 435_GO | CA870009 | AMARELO MIUDO | 1999 | GO |
| 436_GO | CA870011 | BREJEIRO BRANCO | 1999 | GO |
| 437_GO | CA870012 | CARIOQUINHA AMARELO | 1999 | GO |
| 438_GO | CA870014 | CARIOQUINHA | 2000 | GO |
| 439_GO | CA870015 | AGULHA FERRUJO | 2000 | GO |
| 440_GO | CA870018 | BICO PRETO | 1996 | GO |
| 441_GO | CA870019 | BICO PRETO GRAUDO | 1996 | GO |
| 442_GO | CA870020 | BRANCAO | 2000 | GO |
| 443_GO | CA870024 | ARROZ PRECOSTA | 1999 | GO |
| 444_GO | CA870025 | 3 MESES AMARELO | 2000 | GO |
| 445_GO | CA870027 | ARROZ GUAIRA | 1996 | GO |
| 446_GO | CA870029 | BRANCO AGULHA | 1994 | GO |
| 447_GO | CA870032 | GUAIRA AMARELO | 2000 | GO |
| 449_GO | CA870045 | BEIRA CAMPO | 2000 | GO |
| 450_GO | CA870047 | FERRAO PRETO | 2000 | GO |
| 451_GO | CA870048 | PRETO/PRATAO PRECOCE | 2000 | GO |
| 452_GO | CA870050 | BRANCO 4 MESES | 2000 | GO |
| 453_GO | CA870055 | 3 MESES ANTIGO | 2000 | GO |
| 454_GO | CA870056 | CARIOCA/RABO DE CARNEIRO | 2000 | GO |
| 455_GO | CA870057 | BRANCO COMPRIDO | 2000 | GO |
| 456_GO | CA870064 | ARROZ SANTA INÊS | 2000 | GO |
| 457_GO | CA870066 | 3 MESES PRECOCE | 2000 | GO |
| 458_GO | CA870068 | ARROZ COMPRIDO | 1994 | GO |
| 459_GO | CA870069 | 3 MESES | 2000 | GO |
| 460_GO | CA870071 | CARIOCA | 2000 | GO |
| 461_GO | CA870073 | BRANCO ANTIGO | 2000 | GO |
| 462_GO | CA870074 | AMARELAO/4 MESES | 2000 | GO |
| 463_GO | CA870077 | AMARELAO/BICO CLARO | 1999 | GO |
| 464_GO | CA870078 | MESES BRANCO/3 M AMARELO | 1999 | GO |
| 465_GO | CA870079 | FERRAO PRETO/4 MESES | 2000 | GO |
| 467_GO | CA870084 | PRETO | 1996 | GO |
| 468_MG | CA870090 | ARROZ AMARELO | 2000 | MG |
| 469_MG | CA870092 | BRANQUINHO | 2000 | MG |
| 470_MG | CA870098 | 60 DIAS | 2000 | MG |
| 471_MG | CA870100 | RABO DE CARNEIRO | 2000 | MG |
| 472_MG | CA870101 | CURTINHO | 2000 | MG |
| 473_MG | CA870103 | ARROZ DE CAPINÓPOLIS | 2000 | MG |
| 474_MG | CA870105 | AMARELO | 2000 | MG |
| 475_MG | CA870107 | ARROZ CURTO | 2000 | MG |
| 476_MG | CA870109 | 4 MESES ANTIGO | 2000 | MG |
| 477_MG | CA870112 | BRANCO | 2000 | MG |
| 478_MG | CA870116 | AMARELAO 3 MESES | 2000 | MG |
| 479_MG | CA870117 | 4 MESES CASCUDO | 2000 | MG |
| 481_MG | CA870119 | 3 MESES CURTO | 2000 | MG |
| 482_MG | CA870120 | AGULHINHA BRANCO | 2000 | MG |
| 483_MG | CA870121 | ARROZ FARTURA | 2000 | MG |
| 484_MG | CA870129 | FERRAO /4 MESES | 2000 | MG |
| 485_GO | CA870137 | GUAIRA AMARELO E BRANCO | 1999 | GO |
| 486_GO | CA870139 | NOVENTINHA | 1999 | GO |
| 487_GO | CA870144 | BICO ROXO | 2000 | GO |
| 488_GO | CA870149 | 4 MESES | 2000 | GO |
| 489_GO | CA870150 | RABO DE OVELHA | 1999 | GO |
| 490_GO | CA870152 | CAROLINA | 2000 | GO |
| 491_GO | CA870153 | ARROZ DA TERRA | 1999 | GO |
| 492_MG | CA870160 | CASCA BRANCA | 1999 | MG |
| 493_MG | CA870162 | ARROZ TRES MESES | 1999 | MG |
| 494_MG | CA870163 | ARROZ 100 DIAS | 1999 | MG |
| 495_MG | CA870164 | ARROZ CARIOCA | 2000 | MG |
| 496_MG | CA870172 | MERUIM DOURADO | 1999 | MG |
| 497_MG | CA870175 | ARROZ DO MARANHAO | 2000 | MG |
| 498_MG | CA870177 | ARROZ ROXO OU CAQUI | 1999 | MG |
| 507_MT | CA880006 | ARROZ AVIAO | 1989 | MT |
| 508_MT | CA880009 | 4 MESES LEGITINO | 1996 | MT |
| 509_MT | CA880010 | LEGITINO | 1996 | MT |
| 510_MT | CA880011 | ARROZ DA REGIAO | 1996 | MT |
| 511_MT | CA880013 | PACUZINHO | 1989 | MT |
| 512_MT | CA880015 | ARROZ RAJADINHO | 1996 | MT |
| 513_MT | CA880016 | AGULHA 4 MESES | 1996 | MT |
| 514_MT | CA880019 | AGULHA | 1996 | MT |
| 515_MT | CA880022 | MIUDO | 1996 | MT |
| 516_MT | CA880024 | RAJADO | 2000 | MT |
| 517_MT | CA880030 | AMARELAO B.PRETO/3 MESES | 1989 | MT |
| 518_MT | CA880031 | 3 MESES COMPRIDO | 1994 | MT |
| 521_MT | CA880044 | AGULINHA | 1996 | MT |
| 522_MT | CA880046 | FERRAO PRETO CANA ROXA | 2000 | MT |
| 523_MT | CA880047 | AGULHA BRANCO 4 MESES | 1989 | MT |
| 524_MT | CA880048 | 3 MESES BRANCO | 1996 | MT |
| 525_MT | CA880049 | BRANCO C/ ARISTA | 1996 | MT |
| 526_MT | CA880051 | BICO PRETO ACHATADINHO | 1989 | MT |
| 527_MT | CA880053 | BICO GANGA CANA ROXA | 2000 | MT |
| 528_MT | CA880056 | 3 MESES DE ABRIL | 1989 | MT |
| 529_MT | CA880057 | ARROZ DE 3 MESES | 2000 | MT |
| 530_MT | CA880058 | CURTO 4 MESES | 1989 | MT |
| 531_MT | CA880060 | BICO GANGA | 1996 | MT |
| 532_MT | CA880062 | AMARELAO 60 DIAS | 1995 | MT |
| 534_MT | CA880066 | BICO PRETO CANA ROXA | 1989 | MT |
| 535_MT | CA880067 | AGULHINHA 3 MESES | 1989 | MT |
| 536_MT | CA880073 | CATETO | 2000 | MT |
| 538_MT | CA880075 | AGULHA DA TERRA | 1989 | MT |
| 540_MT | CA880080 | ARROZ 10 ANOS | 1989 | MT |
| 541_MT | CA880081 | CATETAO | 1996 | MT |
| 542_MT | CA880083 | ARROZ PRETO | 1996 | MT |
| 543_MT | CA880085 | CATETINHO | 1989 | MT |
| 545_MT | CA880088 | CACHO GRANDE | 1989 | MT |
| 546_MT | CA880093 | ARROZ PIRIQUITO | 1989 | MT |
| 547_MT | CA880099 | ARROZ CARIJO | 1989 | MT |
| 549_MG | CA890001 | ARROZ ANAO | 2000 | MG |
| 550_GO | CA890003 | PRATINHA | 2000 | GO |
| 551_TO | CA890005 | ARROZ ANAOZINHO | 2000 | TO |
| 552_TO | CA890006 | ARROZ BEIRA CAMPO 4 MESES | 2000 | TO |
| 553_GO | CA910001 | ARROZ TORTO | 1996 | GO |
| 554_PB | CA910002 | CAQUI | 1996 | PB |
| 555_TO | CA930001 | BACABA OU ARROZ ROXO | 2000 | TO |
| 558_GO | CA930004 | PARAZINHO | 2000 | GO |
| 559_RS | CA940003 | JAPONES | 1996 | RS |
| 560_GO | CA940010 | ZEBRINHA OU 60 DIAS | 1997 | GO |
| 561_GO | CA950002 | AGULHINHA DOURADO IPAMERI | 1997 | GO |
| 562_GO | CA950003 | AGULHINHA IPAMERI PALHA | 1999 | GO |
| 563_GO | CA950006 | FOICINHA | 1996 | GO |
| 564_MT | CA950007 | AGULHINHA 5 MESES BRANCO | 1997 | MT |
| 565_PR | CA950011 | CATETO AMARELO | 1999 | PR |
| 568_AL | CA960001 | CAIANINHA | 2000 | AL |
| 569_AL | CA960006 | CAROLINDA BRANCO | 2000 | AL |
| 570_AL | CA960008 | CAIANA GRANDE | 2000 | AL |
| 571_AL | CA960010 | 90 DIAS | 2000 | AL |
| 572_AL | CA960011 | CAIANA PEQUENO | 2000 | AL |
| 573_AL | CA960017 | AMARELINHO | 2000 | AL |
| 574_AL | CA960020 | CANARINHO | 2000 | AL |
| 575_AL | CA960027 | CHATINHO | 2000 | AL |
| 576_AL | CA960029 | ANAO | 2000 | AL |
| 577_AL | CA960030 | ANAOZINHO | 2000 | AL |
| 578_AL | CA960033 | JAQUARI | 2000 | AL |
| 579_AL | CA960036 | MINEIRO | 2000 | AL |
| 580_AL | CA960040 | PAULISTINHA | 2000 | AL |
| 581_AC | CA970009 | BICO PRETO/ PRODUTOR | 2000 | AC |
| 582_MA | CA970012 | AGULHINHA VERMELHO | 2000 | MA |
| 585_MA | CA980005 | ARROZ TORO GRAUDO | 2000 | MA |
| 586_MA | CA980006 | ARROZ TORO MIUDO | 2000 | MA |
| 587_MA | CA980008 | ARROZ AGULHA | 2000 | MA |
| 588_MA | CA980009 | ARROZ PALHA MURCHA | 2000 | MA |
| 589_MA | CA980011 | ARROZ BURITI | 2000 | MA |
| 590_MA | CA980013 | ARROZ CANA BRANCA | 2000 | MA |
| 591_MA | CA980014 | ARROZ CANA ROXA | 2000 | MA |
| 592_MA | CA980015 | ARROZAO | 2000 | MA |
| 593_MA | CA980016 | ARROZ BACABA | 2000 | MA |
| 594_MA | CA980017 | ARROZ LAJAO | 2000 | MA |
| 595_MA | CA980018 | ARROZ VERMELHO | 2000 | MA |
| 596_MA | CA980019 | ARROZ GOIANO | 2000 | MA |
| 597_MA | CA980020 | ARROZ LIGEIRO BRANCO | 2000 | MA |
| 598_MA | CA980021 | ARROZ LIGEIRO VERMELHO | 2000 | MA |
| 599_MA | CA980022 | ARROZ COMUM BRANCO | 2000 | MA |
| 600_MA | CA980023 | ARROZ CANELA DE FERRO | 2000 | MA |
| 603_MA | CA980026 | ARROZ CHATAO | 2000 | MA |
| 604_MA | CA980027 | ARROZ LAJEADO | 2000 | MA |
| 606_MA | CA980029 | ARROZ PINDARE | 2000 | MA |
| 611_PR | CNA0000027 | AGULHA ESAV | 2000 | PR |
| 614_PR | CNA0000499 | BICO BRANCO | 2000 | PR |
| 615_RS | CNA0000777 | CALOURO | 2000 | RS |
| 618_PR | CNA0000810 | CHATAO BRANCO | 2000 | PR |
| 620_PA | CNA0000851 | CAROLINO | 1984 | PA |
| 623_FRC | CNA0000902 | CRISTAL | 2000 | FRC |
| 624_GO | CNA0000937 | CATALAO | 1998 | GO |
| 625_BZL | CNA0000982 | CALORO | 2000 | BZL |
| 626_MG | CNA0001000 | DOURADO PRECOCE | 2000 | MG |
| 627_GO | CNA0001190 | FERNANDES | 2000 | GO |
| 628_FLP | CNA0001193 | FORTUNA | 2000 | FLP |
| 629_GO | CNA0001240 | GUAIRA | 2000 | GO |
| 631_CLB | CNA0001419 | COLOMBIA 1 | 1983 | CLB |
| 632_CLB | CNA0001420 | CARREON | 1985 | CLB |
| 634_AM | CNA0002127 | JAGUARI | 2000 | AM |
| 639_MT | CNA0002523 | MATAO | 2000 | MT |
| 640_SLK | CNA0002524 | MOROBEREKAN | 1985 | SLK |
| 641_SLK | CNA0002580 | MURUMGA | 1997 | SLK |
| 642_BZL | CNA0002741 | PRATAO PRECOCE | 1995 | BZL |
| 643_AM | CNA0002746 | PINGO D'AGUA | 2000 | AM |
| 644_PR | CNA0002773 | PRATAO URBELANDIA | 2000 | PR |
| 645_PR | CNA0002774 | PRATAO TIPO GUEDES | 2000 | PR |
| 646_MT | CNA0002825 | PEROLA DE CAMPINAS | 2000 | MT |
| 648_PR | CNA0002836 | PEROLA | 2000 | PR |
| 649_FLP | CNA0002844 | PALAWAN | 1983 | FLP |
| 651_SC | CNA0002862 | PRECOCINHO | 2000 | SC |
| 652_MT | CNA0002865 | QUATRO MESES | 2000 | MT |
| 653_PR | CNA0002872 | RIZOTO | 1995 | PR |
| 654_MT | CNA0002878 | REXORO | 1995 | MT |
| 655_NE | CNA0003056 | TAPURIPA | 2000 | NE |
| 656_SP | CNA0003275 | 64 DIAS | 2000 | SP |
| 657_MG | CNA0003487 | LAMBARI | 1995 | MG |
| 659_GO | CNA0004200 | TRÊS MESES AMARELO | 2000 | GO |
| 660_FLP | CNA0004308 | CAROLINA SP.407 | 1983 | FLP |
| 661_FLP | CNA0004319 | SALUMPIKIT | 1983 | FLP |
| 662_CLB | CNA0004482 | BRASILEIRO | 1983 | CLB |
| 663_CLB | CNA0004485 | IGUAPE CATETO | 1983 | CLB |
| 664_CLB | CNA0004487 | MAKOUTA | 1983 | CLB |
| 666_CLB | CNA0004503 | CHILENO | 1983 | CLB |
| 667_CLB | CNA0004510 | PIEDAD | 1983 | CLB |
| 668_CLB | CNA0004546 | CHATO RAYADO | 1983 | CLB |
| 669_CLB | CNA0004560 | DONATO | 1983 | CLB |
| 670_CLB | CNA0004573 | CANILLA | 1983 | CLB |
| 673_CLB | CNA0004600 | CANUTO | 1983 | CLB |
| 674_CLB | CNA0004601 | LIGERITO | 1983 | CLB |
| 676_CLB | CNA0004618 | EMBARRENADO | 1983 | CLB |
| 677_CLB | CNA0004623 | PICO NEGRO | 1983 | CLB |
| 678_CLB | CNA0004627 | AZUCENA | 1983 | CLB |
| 680_CLB | CNA0004637 | RABO DE YEGUA | 1983 | CLB |
| 681_CLB | CNA0004647 | CHONGONENO | 1983 | CLB |
| 682_CLB | CNA0004762 | PANA | 1983 | CLB |
| 683_FLP | CNA0004763 | MIRITI | 1983 | FLP |
| 685_GO | CNA0004799 | JARAGUA | 1983 | GO |
| 687_MG | CNA0004830 | ARROZ DE 100 DIAS | 1983 | MG |
| 688_GO | CNA0005178 | ARROZ DE CAMPO | 2000 | GO |
| 689_GO | CNA0005564 | ARROZ PIAUI | 2000 | GO |
| 690_GO | CNA0005584 | ARROZ DE REVENDA | 2000 | GO |
| 691_GO | CNA0005659 | ARROZ LIGEIRO | 1985 | GO |
| 692_GO | CNA0005667 | BEIRA CAMPO DOURADO | 2000 | GO |
| 694_GO | CNA0005676 | AGULHINHA OU PARANAZINHO | 1998 | GO |
| 695_GO | CNA0005677 | JARAGUA PONTA PRETA | 2000 | GO |
| 699_GO | CNA0008429 | AGULHINHA IPAMERI | 1996 | GO |

*Abbreviations for collection sites: AC: Acre; AL: Alagoas; AM: Amazonas; BA: Bahia; BZL: Brazil; CE: Ceara; CLB: Colombia; ES: Espirito Santo; FLP: Philippines; FRC: France; GO: Goias; MA: Maranhao; MG: Minas Gerais; MS: Mato Grosso do Sul; MT: Mato Grosso; NE: Northeastern Brazil; PA: Para; PB: Paraiba; PI: Piaui; PR: Parana; RJ: Rio de Janeiro; RO: Rondonia; RR: Roraima; RS: Rio Grande do Sul; SC: Santa Catarina; SE: Sergipe; SLK: Sri Lanka; SP: Sao Paulo; TO: Tocantins
